# Unveiling the uncommon: diagnostic journey of camurati-engelmann disease in a pediatric patient

**DOI:** 10.1186/s12969-024-01016-9

**Published:** 2024-10-08

**Authors:** Ayşenur Alkaya, Adalet Elçin Yıldız, Esra Bağlan, Semanur Özdel

**Affiliations:** 1 Department of Pediatric Rheumatology, Ankara Etlik City Hospital, Ankara, Turkey; 2 Department of Pediatric Radiology, Hacettepe University, Ankara, Turkey

**Keywords:** Camurati Engelmann, TGFB-1, Gait disturbance

## Abstract

**Background:**

Camurati-Engelmann disease (CED), also known as progressive diaphyseal dysplasia, is a rare genetic disorder characterized by abnormal thickening of the long bones’ diaphysis. This condition is caused by mutations in the transforming growth factor beta-1 (TGFB-1) gene and is typically inherited in an autosomal dominant pattern. Patients with CED often present with symptoms such as chronic bone pain, muscle weakness, fatigue, and difficulty walking.

**Case presentation:**

We report a 30-month-old boy who presented with gait abnormality. Initially, toxic synovitis was considered, and non-steroidal anti-inflammatory (NSAİ) treatment was administered. The patient did not respond to NSAİ treatment. Direct radiographs showed diaphyseal thickening, especially in the long bones. Radiologically, CED was suspected, and clinical exome sequencing identified a TGFB-1: c1121C > G (Pro374Arg) heterozygous mutation, which was interpreted as a possible pathogenic variant for CED. A clinical, radiologic, and genetic diagnosis of CED was made.

**Conclusion:**

Due to its rarity and variable clinical presentation, the diagnosis of CED can be challenging and often requires a high index of suspicion. Early and accurate diagnosis is crucial for managing symptoms and improving patients’ quality of life.

## Background

Camurati-Engelmann disease (CED), also known as progressive diaphyseal dysplasia, is a rare genetic disorder characterized by abnormal thickening of the long bones’ diaphysis. CED is an autosomal dominant (OD) inherited disorder resulting from increased osteoblastic activity due to a gain of function mutation in the transforming growth factor beta-1 (TGFB-1) gene on chromosome 19q13 [1, 2]. Proximal muscle weakness, easy fatigue, bone pain especially affecting the lower extremities, waddling gait and marfanoid appearance are the most common clinical features of CED [3]. In cases in which CED is clinically suspected, demonstration of increased radioactivity on bone scintigraphy and periosteal thickening on direct radiography in the early period is helpful for the diagnosis [4]. Definitive diagnosis is made when a genetic mutation is demonstrated. Clinical exome sequencing and whole exome sequencing can be used [3]. Herein we report a 30-month-old presenting with gait abnormality and was subsequently diagnosed with CED.

## Case report

**Fig. 1:**
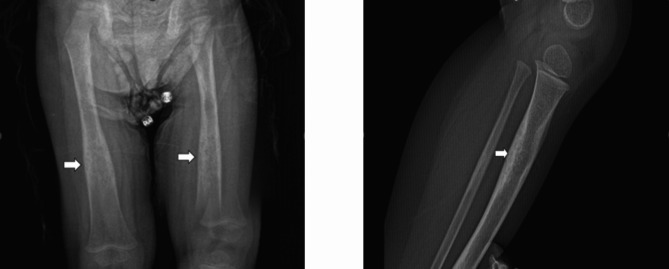
Radiography of the lower limbs shows cortical thickening of both femurs and tibia

A currently 30-month-old boy was referred to our pediatric rheumatology outpatient clinic due to gait disturbance. The patient, who walked for the first time at the age of 18 months, had complaints of pain in both legs and inability to run since 24 months of age. On physical examination, deep tendon reflexes were normoactive in the upper and lower extremities and no muscle weakness was found. The patient had painful hip movements, which were prominent on the left side, and a wide based antalgic gait was remarkable. Laboratory tests revealed white blood cell: 11.160 /µL, hemoglobin: 11.5 g/dl, platelet: 350.000 /µL, creatine kinase: 79 U/L, calcium: 9.8 mg/dl, c-reactive protein: 0.21 mg/L, erythrocyte sedimentation rate: 15 mm/h, anti-nuclear antibody (ANA), rheumatoid factor (RF) and HLA-B27 values were negative. Initially, toxic synovitis was considered, and non-steroidal anti-inflammatory (NSAİ) treatment was administered. The patient did not respond to NSAİ treatment. Due to the persistence of the patient’s complaints, a hip magnetic resonance imaging (MRI) performed on the patient resulted normal. The patient subsequently presented to another center at his family own request. A thigh MRI performed there suggested the possibility of Langerhans cell histiocytosis, leading to a bone biopsy being conducted. The bone biopsy was reported as normal. The patient later returned to our hospital. Direct radiographs showed diaphyseal thickening especially in the long bones (Fig. 1). With a preliminary diagnosis of chronic nonbacterial osteomyelitis, a whole-body MRI was performed. This MRI led the radiologists to consider the possibility of CED. Whole body MRI showed hyperostosis and periosteal thickening in multicentric long bones. CED was suspected radiologically and TGFB-1: c1121C > G (Pro374Arg) heterozygous mutation was interpreted as a possible pathogen for CED in clinical exome sequencing. A clinical, radiologic and genetic diagnosis of CED was made. After this diagnosis was made, the patient did not return for further follow-up.

## Discussion and conclusions

CED is a rare and OD inherited genetic disease. Patients most commonly present with complaints including gait disturbance, limb pain and muscle weakness [3]. In a review including 199 patients with CED, bone pain was reported in 86% and waddling gait in 73% [5]. TGFB-1 activation increases osteoblastic differentiation and decreases osteoclastic activity. This increases intramembranous ossification and increases bone mass in the diaphysis of long bones. It leads to hypogonadotropic hypogonadism by suppressing adipogenesis and gonad development. Steroids commonly used in the treatment of CED reverse these effects by inhibiting TGBF-1 activation and suppress inflammation [6]. Losartan, an angiotensin II receptor antagonist, has been reported to reduce bone pain and increase physical activity capacity by inhibiting TGFB-1 activation [7]. Improvement in bone mineral density and onset of puberty have been reported in an 18-year-old CED patient treated with steroids and losartan [6]. The role of bisphosphonates in the treatment of CED is controversial. There are cases reporting increased bone pain and improvement in bone mineral density [4]. Two cases treated with infliximab have been reported in the literature. While a decrease in bone pain and an increase in bone mineral density at the lumbar level were reported in a 46-year-old CED patient who was started on infliximab for ulcerative colitis, worsening of bone pain was reported in another 15-year-old patient who was started on infliximab for Crohn’s disease [8, 9].

CED may be confused with chronic non-bacterial osteomyelitis (CNO) due to widespread multicentric involvement. In CNO with symmetric involvement of long bones, shoulder girdle and pelvic bones, lytic lesions are observed in the metaphysis of bones with increased osteoclastic activity [10].

CED is a progressive disease that starts with diaphyseal involvement of the long bones and progresses to the skull bones. Involvement of the skull bones can lead to neurologic, endocrine, hearing and visual problems. Physical therapy exercises may be required to relieve pain, increase physical capacity and prevent joint contractures. Multidisciplinary follow-up of patients with CED with relevant departments is important for the management of future complications.

## Data Availability

Not applicable.

## Abbreviations

CED: Camurati Engelmann Disease
CNO: Chronic Non-bacterial Osteomyelitis
NSAI: Nonsteroidal Anti-inflammatory
MRI: Magnetic Resonance Imaging
TGFB-1: Transforming Growth Factor Beta-1
